# The Deadly Itch: A Rare but Fatal Case of Norwegian Scabies

**DOI:** 10.7759/cureus.83311

**Published:** 2025-05-01

**Authors:** Noor Sadiq Syed, Harsh AshokBhai Padhiyar, Syed Ali Hassan

**Affiliations:** 1 Emergency Medicine, The Mid Yorkshire National Health Service (NHS) Teaching Trust, Wakefield, GBR; 2 Emergency Medicine, Leeds Teaching Hospitals National Health Service (NHS) Trust, Leeds, GBR

**Keywords:** crusted scabies, hyperkeratotic skin lesions, neglected disease, norwegian scabies, sepsis in scabies

## Abstract

Norwegian (crusted) scabies is a rare but highly contagious variant of scabies that predominantly affects immunocompromised individuals, such as those with diabetes or neurological disorders. The condition is characterised by hyperkeratotic plaques teeming with mites, often leading to secondary bacterial infections and life-threatening complications. We present a fatal case of Norwegian scabies in a 65-year-old male with epilepsy and diabetes, who developed severe skin infestation, staphylococcal bacteremia, and multi-organ failure. Despite receiving aggressive treatment with oral ivermectin, topical permethrin, and IV antibiotics, the patient deteriorated and succumbed to sepsis. This case highlights the importance of early diagnosis, aggressive management, and strict infection control to prevent adverse outcomes.

## Introduction

Scabies is a highly contagious parasitic skin infestation caused by the itch mite *Sarcoptes scabiei* var. *hominis* [1]. It affects individuals of all ages and backgrounds globally, often spreading rapidly in crowded environments such as care homes, hospitals, nurseries, boarding dorms, and shelters. Transmission typically occurs through prolonged skin-to-skin contact, making it a significant public health concern, especially among vulnerable populations.

There are two major clinical variants of scabies: classic scabies, which is the more common form, and crusted scabies, also known as Norwegian scabies [2]. Classic scabies usually presents with intense nocturnal itching and multiple erythematous (red) papules, often excoriated due to scratching [3]. In contrast, Norwegian scabies is a rare but severe form of the disease, characterised by widespread thick crusts, scaling, and hyperkeratotic plaques that may or may not be itchy [4,5]. This form has a much higher mite burden, containing thousands to millions of mites, compared to the 10-15 mites typically found in classic scabies, making it highly infectious and difficult to manage.

Diagnosing Norwegian scabies can be challenging due to its atypical presentation, which can resemble psoriasis, eczema, or other dermatoses [6]. A definitive diagnosis is typically made by identifying mites or eggs under light microscopy.

Crusted scabies is more commonly seen in immunocompromised individuals, such as patients on systemic corticosteroids or chemotherapy, or those with cognitive impairment or severe malnutrition [7]. Due to its high infectivity and potential for rapid spread in healthcare settings, early recognition, prompt treatment, and rigorous infection control measures are crucial.

In this report, we present a rare case of Norwegian scabies in an elderly, immunosuppressed patient that was complicated by sepsis. This case highlights the diagnostic challenges and potentially fatal consequences of delayed identification and treatment. It is, therefore, important to raise awareness of such severe presentations among clinicians of all specialities.

## Case presentation

A 65-year-old male patient presented to the emergency department with progressive skin lesions, confusion, and a recent fall. He had been experiencing a worsening, scaly rash for the past nine months, which his general practitioner initially treated as a fungal infection. His wife also reported gradual weight loss, declining mobility, and worsening cognitive abilities.

**Figure 1:**
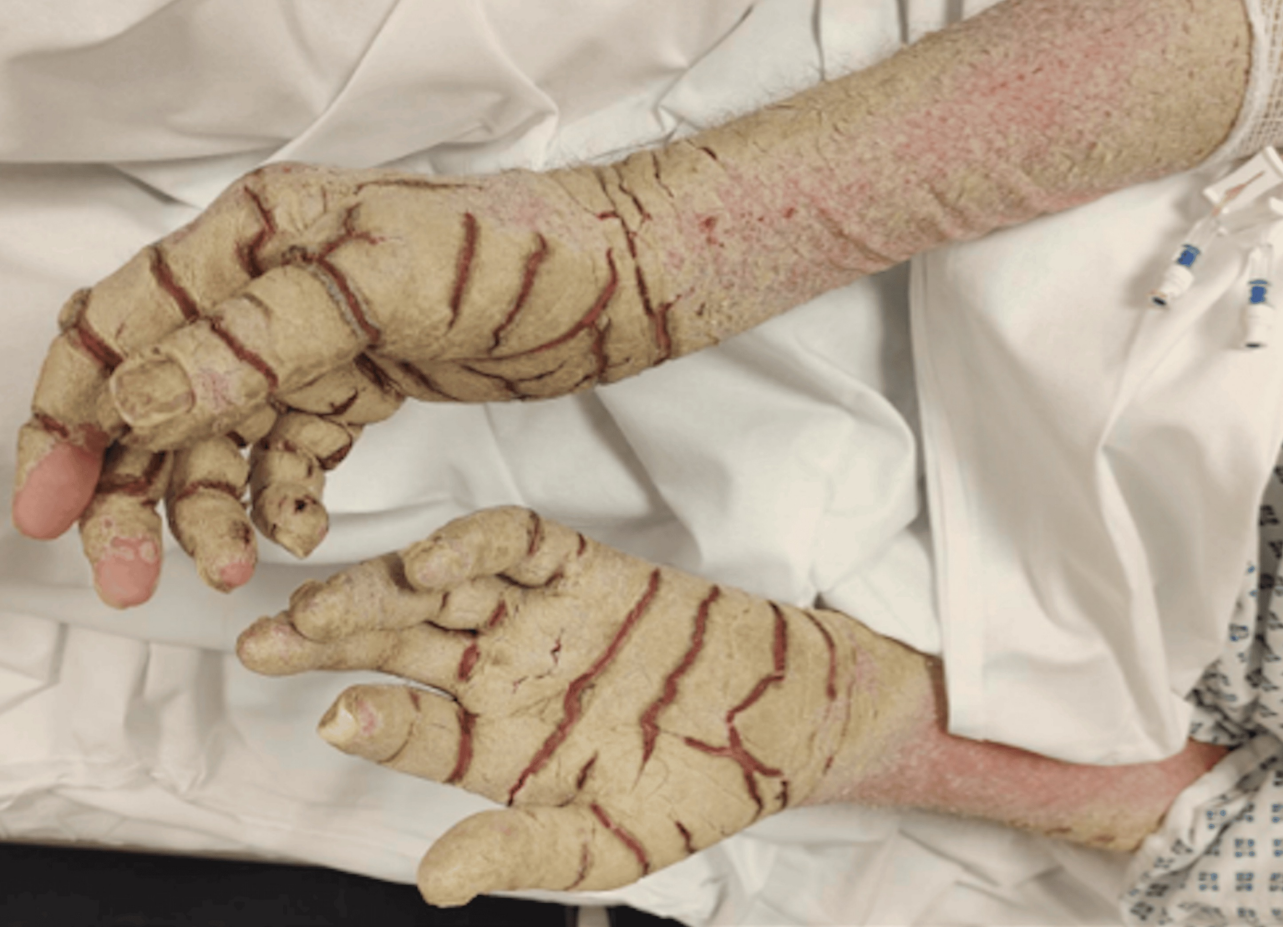
Crusted lesions on the arms and hands

**Figure 2:**
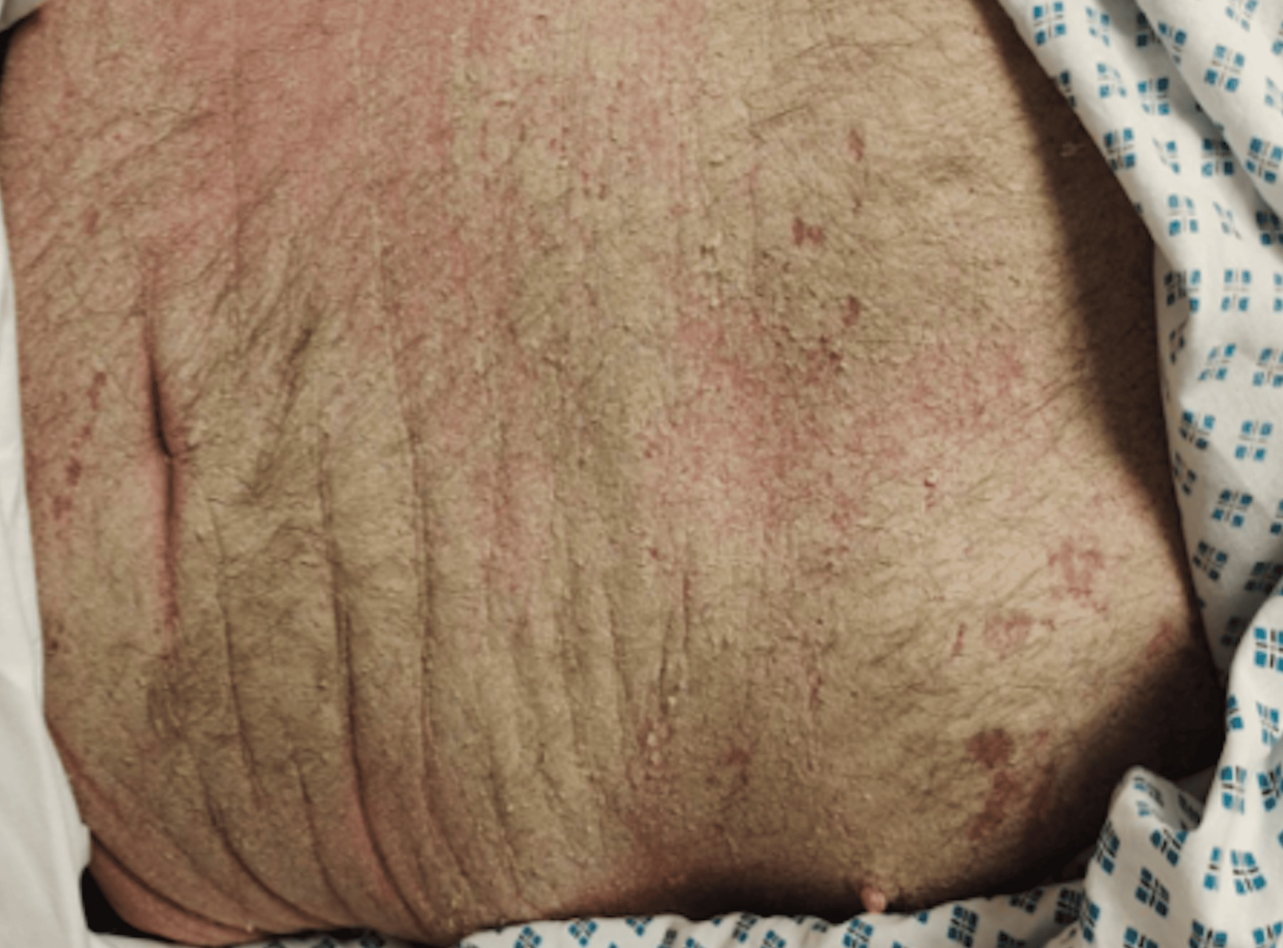
Crusted lesions on the torso

**Figure 3:**
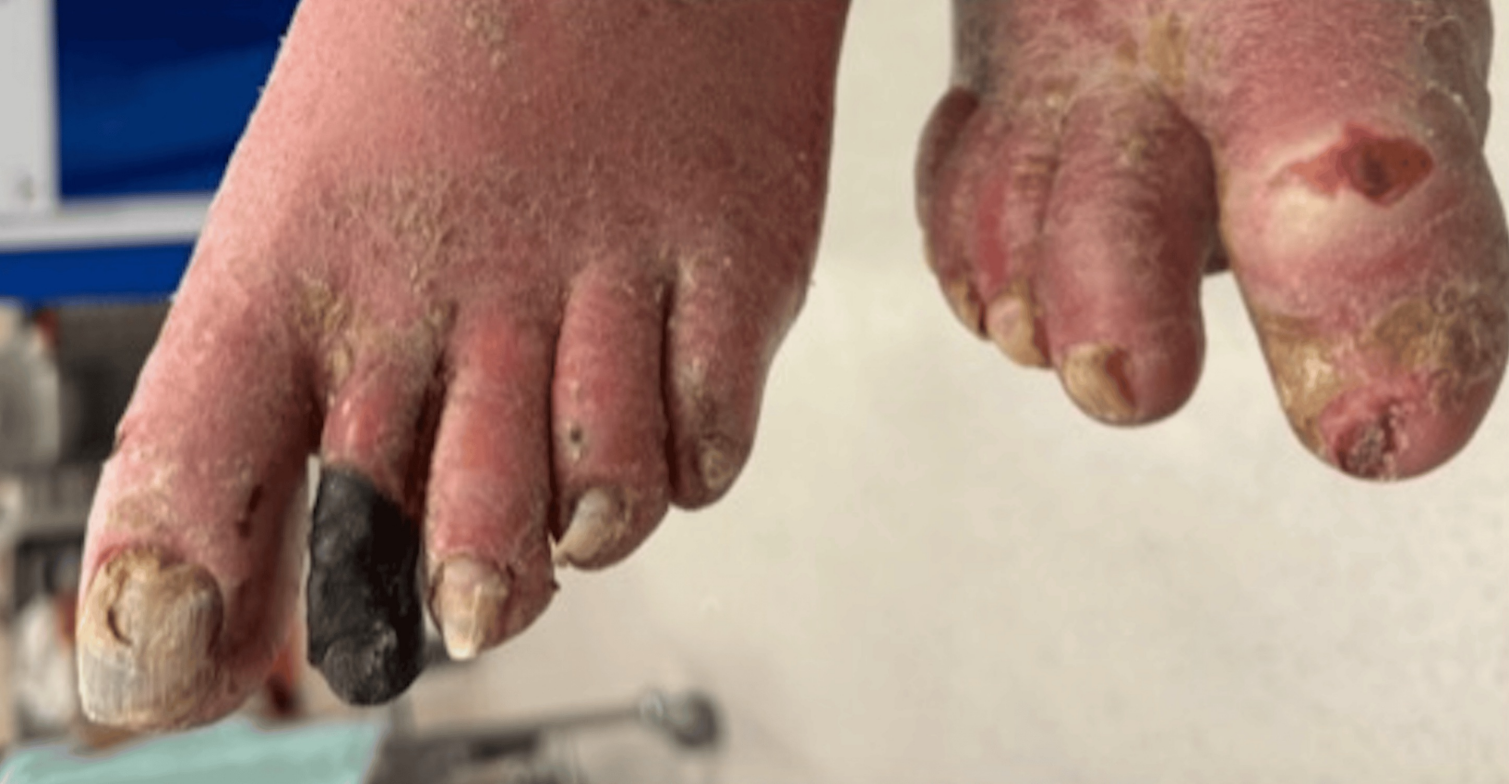
Necrotic left foot

On arrival, his National Early Warning Score was 4 (blood pressure: 85/57 mmHg, heart rate: 80 bpm, Glasgow Coma Scale: 14/15, temperature: 35.9°C, peripheral oxygen saturation: 96%). He had extensive hyperkeratotic plaques and crusting over the hands and arms (Figure 1), torso (Figure 2), neck, and groin. The second toe on the left foot had become necrotic (Figure 3), raising concerns about ischaemia and the risk of secondary infection. The patient reported initial itching but mentioned he never felt the urge to scratch. Given his rapidly declining mental status and haemodynamic instability, he was intubated and transferred to the intensive care unit for further management.

His skin scrapings showed an extensive mite burden, which confirmed Norwegian scabies, and blood cultures were suggestive of *Staphylococcus aureus* and coliform bacteremia. Treatment was initiated based on dermatological input, with oral ivermectin 200 mcg/kg administered on days 1, 2, 8, and 15, and topical permethrin 5% applied daily, along with other dermatological recommendations. He was also started on intravenous tazocin and IV fluids with electrolyte correction. Supportive care included active rewarming for hypothermia, noradrenaline infusion for sepsis-induced hypotension, and wound care for a necrotic toe. Strict infection control strategies were implemented, including the use of personal protective equipment kits for staff and family and terminal room cleaning after each procedure.

Despite aggressive treatment, the patient’s condition deteriorated, and he remained haemodynamically unstable, requiring escalating vasopressor support. Given his poor neurological status and worsening multi-organ dysfunction, a palliative approach was adopted, and a "do not attempt cardiopulmonary resuscitation" decision was made. The patient was extubated on comfort measures and ultimately succumbed to his disease due to septic shock and multi-organ failure.

## Discussion

In our case report, we describe an unusual presentation of Norwegian scabies complicated by sepsis and its implications for delaying diagnosis and treatment. Norwegian scabies poses significant diagnostic and therapeutic challenges, particularly in individuals with neurological impairment or immunosuppression. Had our patient been diagnosed earlier, aggressive therapy might have prevented the complications and mortality.

The association between immunosuppression and crusted scabies is well established, with the most plausible explanation being an inadequate host response to mite proliferation [8]. Numerous hypotheses have been proposed to explain the relationship between cognitive delay and a reduced ability to interpret and express pruritus [9,10], as well as subtle immune system abnormalities observed in the elderly population [10].

Various other risk factors have also been identified for the occurrence of this disease, including a history of alcohol and/or drug abuse, corticosteroid use, cancer chemotherapy (e.g., methotrexate or cyclosporine, representing non-corticosteroid immunosuppression), HIV/AIDS, and type 2 diabetes mellitus.

Associated conditions with Norwegian scabies

Viral and Bacterial Infections

Underlying bacterial or viral infections are commonly observed in patients with crusted scabies. Studies have reported a high prevalence of these infections among such patients [11]. Another common immunosuppression-related viral infection is human T-lymphotropic virus 1 (HTLV-1), which was identified in 8.5% of cases in a large case series from Brazil and Peru [9,10]. Reported bacterial infections included tuberculosis, leprosy (2.3%), pneumonia, syphilis, and a previous methicillin-resistant *Staphylococcus aureus* infection. These bacterial infections, combined with the patient’s immunocompromised status [8], led to sepsis and multi-organ failure, as was the case with our patient.

Neurological and/or Cognitive Impairment

Many case reports have described patients with Norwegian scabies alongside cognitive and neurological impairments. Among these, Alzheimer's disease was the most common (6.1%), followed by Down syndrome (4.1%). Other neurological conditions included HTLV-1-associated myelopathies, peripheral neuropathy, and spinal/motor nerve damage [11-13].

Skin and Autoimmune Conditions

Crusted scabies is frequently misdiagnosed as other atopic skin conditions, such as dermatitis and psoriasis, as was the case in our patient. However, the presence of these pre-existing conditions was not considered a significant risk factor until it was highlighted in studies by Cebeci et al. [12] and Lee et al. [13]. A history of atopic dermatitis was the most common risk factor, followed by other pre-existing skin conditions such as bullous pemphigoid, dermatomyositis, previous burns, psoriasis, systemic lupus erythematosus, and rheumatoid arthritis.

Additional Conditions

Additional conditions associated with the development of Norwegian scabies include leukemia, transplants (3.4%), cancers (3.5%), and endocrine disorders (0.9%). Among the leukemias, T-cell leukemia is the most common [11].

## Conclusions

Early recognition and prompt dermatological consultation are crucial for timely diagnosis and initiation of appropriate therapy. The combination of oral ivermectin and topical permethrin remains the gold standard treatment for Norwegian scabies. Delayed or inadequate treatment significantly increases the risk of secondary bacterial infections, which can progress to sepsis and multi-organ failure, particularly in vulnerable populations. Although very rare, Norwegian scabies continues to pose a life-threatening risk in immunocompromised or neurologically impaired patients. Clinicians should maintain a high index of suspicion in individuals presenting with widespread crusted skin lesions, especially in the context of underlying neurological or metabolic conditions. In such cases, early intervention with aggressive anti-parasitic therapy, alongside strict infection control measures, is critical to improving patient outcomes and preventing institutional outbreaks. To the best of our knowledge, the rarity and severity of this presentation underscore the importance of reporting such cases to raise awareness, aid early recognition, and reduce mortality in high-risk patient populations.
